# Convolutional Neural Network for Object Detection in Garlic Root Cutting Equipment

**DOI:** 10.3390/foods11152197

**Published:** 2022-07-24

**Authors:** Ke Yang, Baoliang Peng, Fengwei Gu, Yanhua Zhang, Shenying Wang, Zhaoyang Yu, Zhichao Hu

**Affiliations:** 1 Nanjing Institute of Agricultural Mechanization, Ministry of Agriculture and Rural Affairs, Nanjing 210014, China; yk666666yk@126.com (K.Y.); pengbaoliang@caas.cn (B.P.); gufengwei@caas.cn (F.G.); zhangyanhua@caas.cn (Y.Z.); 2 College of Biosystems Engineering and Food Science, Zhejiang University, Hangzhou 310058, China; wangshenying@caas.cn; 3 Key Laboratory of Modern Agricultural Equipment, Ministry of Agriculture and Rural Affairs, Nanjing 210014, China

**Keywords:** convolutional neural network, YOLO, object detection, garlic root cutting, food safety control

## Abstract

Traditional manual garlic root cutting is inefficient and can cause food safety problems. To develop food processing equipment, a novel and accurate object detection method for garlic using deep learning—a convolutional neural network—is proposed in this study. The you-only-look-once (YOLO) algorithm, which is based on lightweight and transfer learning, is the most advanced computer vision method for single large object detection. To detect the bulb, the YOLOv2 model was modified using an inverted residual module and residual structure. The modified model was trained based on images of bulbs with varied brightness, surface attachment, and shape, which enabled sufficient learning of the detector. The optimum minibatches and epochs were obtained by comparing the test results of different training parameters. Research shows that IRM-YOLOv2 is superior to the SqueezeNet, ShuffleNet, and YOLOv2 models of classical neural networks, as well as the YOLOv3 and YOLOv4 algorithm models. The confidence score, average accuracy, deviation, standard deviation, detection time, and storage space of IRM-YOLOv2 were 0.98228, 99.2%, 2.819 pixels, 4.153, 0.0356 s, and 24.2 MB, respectively. In addition, this study provides an important reference for the application of the YOLO algorithm in food research.

## 1. Introduction

*Allium sativum* L. (gallic) is a plant food with medicinal value and a long history of planting [1,2,3], with a global harvest area of 1.63 million hectares [4]. In addition to the direct consumption of garlic, garlic oil is extracted on an industrial scale worldwide to meet the needs of the pharmaceutical and food industries [5]. In recent years, the medicinal value of garlic has been continuously developed and promoted. Its industrial products have been added to animal feed as broad-spectrum antibacterial agents, which have improved the quality of animal food.

Garlic is cut into roots and stems after harvest [6,7] to obtain valuable bulbs. The purpose of root cutting is to improve the commercial value of bulbs and avoid mildew and deterioration of bulbs caused by soil entrained by garlic roots. At present, owing to the lack of automatic equipment, garlic root cutting is mainly completed by manual operation, which has low production efficiency and makes it easy to cut bulbs, resulting in pathogen invasion and food safety problems [8,9,10]. The cut bulbs are susceptible to infection by pathogenic bacteria, which produce highly toxic mycotoxins that have a negative impact on human health [11]. To improve production efficiency and control food safety, there is an urgent need to automate garlic treatment. Due to the great differences in the individual conditions of garlic, the biggest difficulty faced by the research is how to avoid cutting bulbs.

In recent years, the application of machine learning in food safety control has increased. Results show that most studies have applied Bayesian networks, neural networks, or support vector machines [12]. Using machine learning in the field of food processing helps reduce processing time and ensures higher product quality [13]. The use of a support vector machine classifier and near-infrared spectroscopy to predict the country of origin of white asparagus has high accuracy [14]. Egg classification using a convolutional neural network (CNN) is an advanced and accurate computer vision method [15]. Through the use of a deep learning model based on ShuffleNet, the performance of carrot surface-defect detection was excellent in terms of detection accuracy and time efficiency [16]. However, a simple classification detector could not meet the requirements of this study.

Garlic root cutting requires that the detector not only has the ability to classify but also achieve position detection. The accuracy and reliability of position detection determine not only the quality of root cutting but also the value of the root cutting equipment. In addition, to adapt to a continuous industrial environment, detection must occur in a timely manner. It can be seen that garlic root cutting requires very high detector performance, which concurrently requires high accuracy, speed, and reliability.

CNNs use computational models composed of multiple processing layers to learn abstract data representations and are the latest technological advancement in object detection [17]. An integrated learning approach based on CNN estimators applied to the determination of infant formula adulterants yielded better regression performance [18]. Regions with Convolution Neural Network features (R-CNNs) were the first two-phase algorithm object detection model [19]. With the advent of the single-shot multibox detector (SSD) [20] and you-only-look-once (YOLO) algorithm [21], the one-stage algorithm model for object detection has developed rapidly. Common one-stage algorithm models include the SSD, RatinaNet [22], YOLO, and LRF [23]. A first-stage algorithm does not need to extract the area of interest from the image and only uses the detection head to classify and locate within the feature map. Therefore, this type of algorithm has a high detection speed. Two-stage algorithms include the R-CNN, fast R-CNN [24], and faster R-CNN [25]. This type of algorithm first classifies the foreground and background in one phase, then selects the area of interest, and then proceeds to the second phase to perform detection and location. YOLOv2 detection is faster and more accurate than faster R-CNN and can be run at different resolutions by dynamic adjustment [26]. In zooplankton detection, the improved YOLOv3 detection accuracy is higher than that of the faster R-CNN, and the detection speed is much higher [27]. YOLOv4 has a higher detection accuracy than that of SSD, LRF, Faster R-CNN, and RatinaNet [28], as determined by comparing the MSCOCO datasets. YOLOv2, YOLOv3, and YOLOv4 are the three versions of the YOLO algorithm. Currently, the application of the YOLO algorithm in food research is in its infancy.

An object detector-based bee colony health status monitoring method with online measurement and processing potential has been developed using YOLO and SSD [29]. A mobile vision-based food grading evaluation system was proposed using the YOLOv3 model to overcome the challenge of detecting and outputting small defective areas of bananas [30]. Using the Yolo model to detect mold on food surfaces was new research [31]. The use of the YOLO algorithm simplifies the tedious process of cherry quality inspection and improves the speed and accuracy of inspection [32].

To the best of our knowledge, our team is the first to develop a YOLO object detection system for garlic root cutting. This study was based on our previous research [33]. In this study, an innovative quantitative analysis method for image brightness in datasets is proposed. YOLOv2 was improved using a lightweight module, which makes the advantages of YOLOv2 for single large object detection more significant [34]. In addition, the reliability of the YOLO algorithm detection was examined. Therefore, this study provides an important reference for the application of the YOLO algorithm in the food research field.

## 2. Materials and Methods

### 2.1. Materials

To conduct this research, field excavation experiments were performed to ensure that the garlic plant status was consistent with manual harvest. All garlic plants were obtained from a garlic planting base in Sheyang County, Nanjing Institute of Agricultural Mechanization, Ministry of Agriculture and Rural Areas. Garlic planting in Sheyang has a long history and covers a large area. In the field test, garlic plants were excavated for no longer than 2 h; therefore, the soil on the bulbs and garlic roots was not required to be cleared, and testing was performed directly to preserve the original state of the sample. Garlic root cutting is the primary processing of food, and soil removal is not usually performed before cutting the roots. The original state of the sample was kept to make the algorithm of the study practically usable. Additionally, to verify that the studied object detection algorithm can be used properly in the most difficult detection situation. In total, 540 garlic plants were excavated in this study. Garlic strains were randomly obtained and tested during the experiment. Field tests were conducted in May 2021 with garlic from a field located at 33°51′56″ N, 120°13′49″ E.

### 2.2. Image Acquisition

The industrial camera used in this study was a Minsvision MS-UB500C (Minsvision, Shen Zhen, China), with a sensitivity of 1.76 V/lux, exposure time range of 0.058–905 ms, pixel depth of 12 bits, pixel size of 2.2 × 2.2 μm, and 5 million effective pixels. During the research, the optical axis of the industrial camera was perpendicular to the background plate and kept horizontal. For a clear view of the relative dimensions, a 5 × 5 mm grid was attached to the background plate.

**Figure 1:**
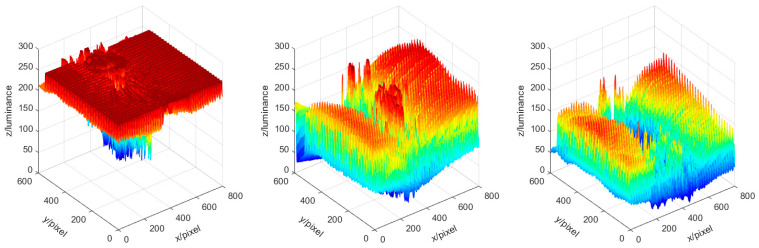

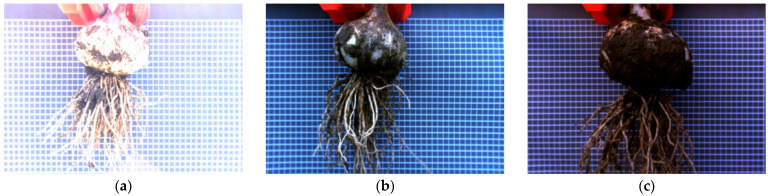
Diagram of brightness level differences. The average Y values are (**a**) 224.4, (**b**) 100.5, and (**c**) 58.7.

As early garlic harvest will depress yields and late bulbs will crack naturally, reducing sales revenue, the appropriate harvest period for garlic is only approximately seven days per year. It can be seen that garlic has a short harvest period and a large amount of harvest work. Considering the practical performance, garlic roots are cut at harvest, resulting in the highest efficiency. Therefore, the equipment used for garlic root cutting must be able to operate under various sunlight conditions, and the images collected must have different brightness values. To quantify brightness, the digital image was converted from RGB to YUV color space [35]. Here, Y represents the brightness information of the image, and the difference in Y values between images with varied brightness is shown in Figure 1.

**Figure 2:**
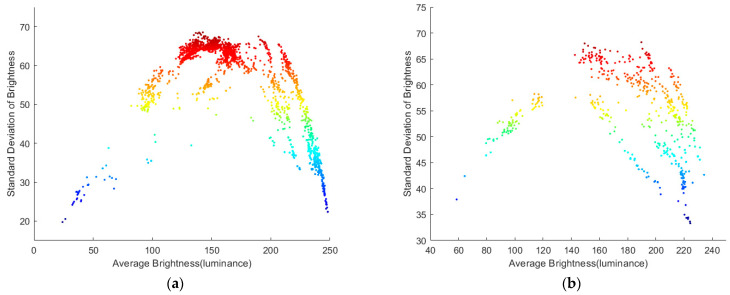
Distribution of image brightness of the datasets: (**a**) training data and (**b**) test data.

Image acquisition takes 3 days, from 8:00 to 19:00. A total of 2500 images were collected without duplication. For the test and training data, 500 and 2000 images, respectively, were randomly selected. The resolution of each image was 800 × 600 pixels. The brightness distribution of the training and test data in the YUV color space is shown in Figure 2. Each point in the point cloud of Figure 2a,b represents an image, and the horizontal and vertical coordinates represent the average and standard deviation of the brightness of each pixel in the represented images. As can be observed from Figure 2, the average range of the training and test data image brightness is between 0 and 255, and the brightness distribution of the training and test data image is similar, covering a wide range.

For the training of the detector, the training data were divided into a training set (1400 images) and a validation set (600 images) in a ratio of 7:3.

### 2.3. Data Annotations

To reduce the computation cost, the image resolution was adjusted before labeling the data to 224 × 168 pixels. LabelImg [36] was used to label the objects in the training set. In this study, the bulbs were labeled as objects. When labeling, we avoided the border of the label box across the bulb and required that the bulb cover as much area as possible in the label box.

### 2.4. Estimate Anchor Boxes

**Figure 3:**
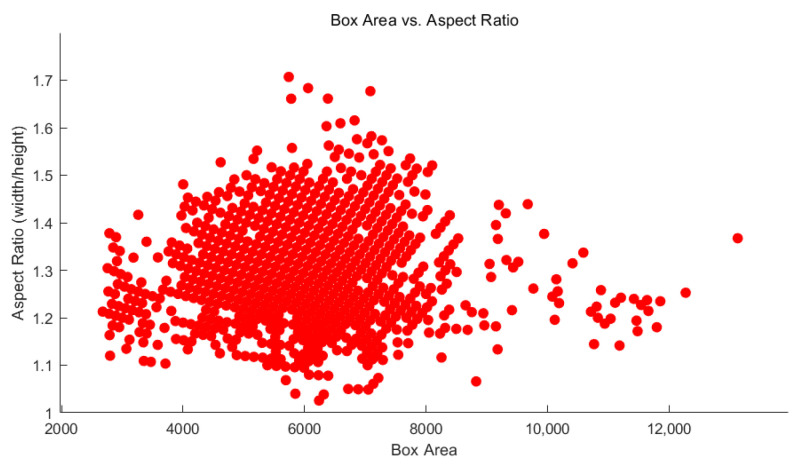
Distribution of training data.

In YOLOv2, the label box of the bulb is called the ground-truth box. The anchor box is the most frequently occurring rectangular box obtained by clustering the length and width of all ground truth boxes in the training set using the K-means clustering algorithm [37]. Estimating the number of anchor boxes is an important step in producing high-performance detectors [38]. The number of anchors is a hyperparameter that affects the efficiency and accuracy of a detector. To better understand the ground truth box in the dataset, the training data are visualized, as shown in Figure 3. The objects in Figure 3 are scattered, and it was difficult to select the anchor box manually.

The intersection over union (IoU), also known as the Jaccard index, is the most popular evaluation metric for tasks such as segmentation, object detection, and tracking. The IoU explores the intersection area I or union area U and is then computed as follows:

(1) $$\mathrm{IoU}=\frac{\left|A\cap B\right|}{\left|A\cup B\right|}=\frac{\left|I\right|}{\left|U\right|}$$

**Figure 4:**
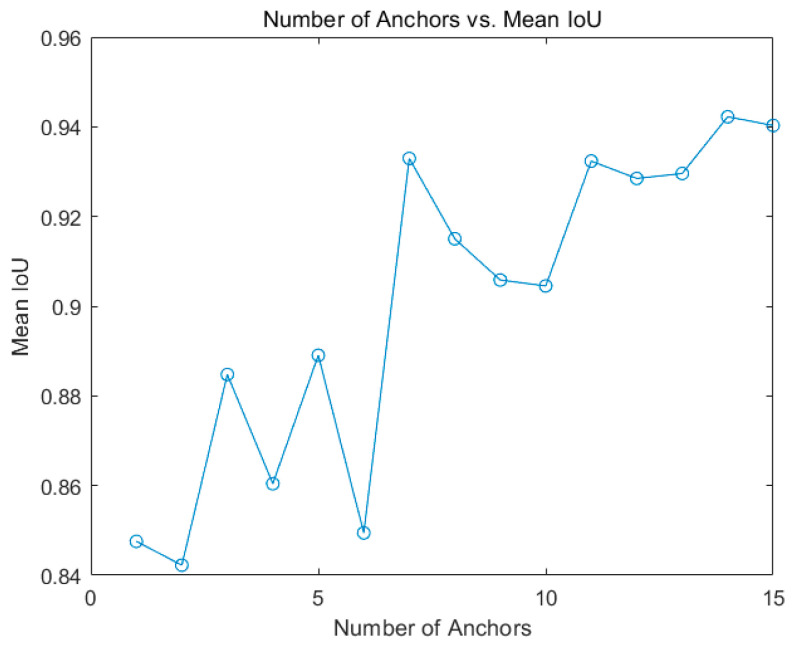
Mean IoU of number of anchors for garlic images.

If the two boxes completely overlap, IoU is 1; if they do not intersect at all, IoU is 0. The mean IOU was used as a measure of the quality of each cluster group [26]. When the number of anchor boxes is seven (mean IOU = 0.9363), the mean IOU reaches a maximum value. As shown in Figure 4, increasing the number of anchor boxes can improve the mean IOU. However, using more anchor boxes in the process of detector training and recognition increases the computational cost and leads to overfitting, which reduces the performance of the detector. Therefore, seven anchor boxes were selected in this study.

### 2.5. Proposed Detector Model

YOLO regards object detection as a regression problem and performs a global analysis of the complete image and all objects in the image. It can perform feature extraction, category prediction, bounding box prediction, confidence estimation, non-maximum suppression, and context reasoning simultaneously, which greatly improves the detection speed [21].

The original YOLOv2 model used DarkNet-19 as the backbone network, with 19 convolutions and 5 largest pooling layers [26]. Darknet-19 utilized a batch normalization (BN) layer, which was used to eliminate the internal covariate displacement caused by changes in the internal node distribution in the deep network so that the deep neural network could be trained faster [39]. However, the network depth of DarkNet-19 was insufficient to meet the need for learning more comprehensive and detailed image features.

#### 2.5.1. Lightweight CNN

**Figure 5:**
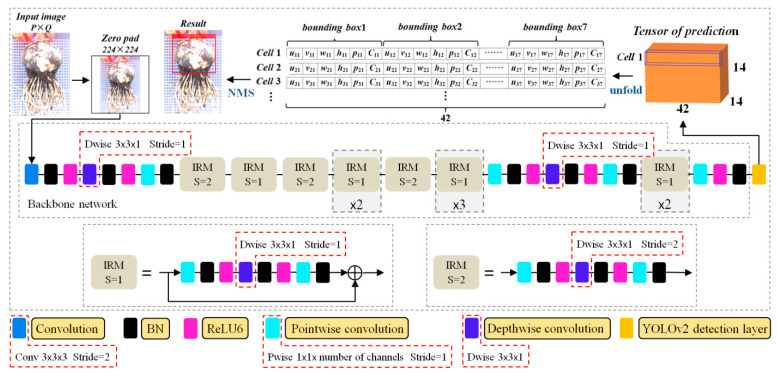
IRM-YOLOv2 model structure.

Based on the original YOLOv2, an IRM-YOLOv2 based on MobileNetV2 was designed, and its structure is shown in Figure 5. An inverted residual module (IRM) is the basic and key unit of MobileNetV2 [40]. A complete IRM begins with one pointwise convolution and then executes one depthwise convolution and another pointwise convolution. Depthwise convolution changes the width and height of the input characteristics map [41]. In the second pointwise convolution, the feature map channels are combined and reduced [42]. In addition, the BN layer attached to each convolution layer can uniformly distribute data and effectively improve the convergence rate of the model [43]. ReLU6, which is robust for low-precision calculations, was used as a nonlinear activation function [44]. Finally, to avoid information loss, the feature map was transferred directly to the next convolution layer [45] without using ReLU6 after the second pointwise convolution and BN layers.

When the string of depthwise convolution in IRM is two, IRM is a linear bottleneck that reduces the size of the feature map. When the stride of the depth-wise convolution in IRM is 1, the IRM is an inverted residual, that is, narrow–wide–narrow. In MobileNetV2, expansion (narrow–wide) and projection (wide–narrow) are achieved with pointwise convolution, as discussed earlier [46,47]. The introduction of an inverted residual effectively inhibits the accumulation of training errors. Deepening the network does not lead to degradation problems and avoids the disappearance of gradients and gradient explosions [48,49,50]. Therefore, after an inverted residual is introduced into the model, the loss of fine features is reduced, and the object detection accuracy is improved [51].

In addition, MobileNet decomposes a standard convolution into depthwise and pointwise convolutions based on the depthwise separable convolution [52]. When depthwise separable convolution works, the input channel is filtered by the depthwise convolution, and the filtering results are linearly combined by pointwise convolution, which avoids the additional combined output generated by the standard convolution layer, thus greatly reducing the calculation amount and model size.

Assume that the input of the standard convolution is *U_F_* × *U_F_* × *M* and the output is *U_F_* × *U_F_* × *N*. *U_F_* is the length and width of the input characteristic map, *M* is the dimension of the input characteristic map, and *N* is the dimension of the output characteristic map. The size of the convolution core of the standard convolution layer is *U_K_* × *U_K_* × *M* × N, where *U_K_* is the length and width of the convolution core. The computational cost of the convolution is calculated as [52]:

Standard convolutions have the computational cost of

(2) $$U_{K}\times U_{K}\times M\times N\times U_{F}\times U_{F}$$

Depthwise convolution has a computational cost of

(3) $$\;U_{K}\times U_{K}\times M\times U_{F}\times U_{F}$$

Depthwise separable convolutions have a cost of

(4) $$U_{K}\times U_{K}\times M\times U_{F}\times U_{F}+M\times N\times U_{F}\times U_{F}$$

By expressing convolution as a two-step process of filtering and combining, we obtained a reduction in the computation of

(5) $$\frac{U_{K}\cdot U_{K}\cdot M\cdot U_{F}\cdot U_{F}+M\cdot N\cdot U_{F}\cdot U_{F}}{U_{K}\cdot U_{K}\cdot M\cdot N\cdot U_{F}\cdot U_{F}}=\frac{1}{N}+\frac{1}{U_{K}^{2}}$$

In this study, 3 × 3 depthwise separable convolutions were used, and thus, the operation of a single convolution was 8–9 times less than that of a standard convolution. A total of 10 depthwise separable convolutions were included in the IRM-YOLOv2.

In addition, IRM-YOLOv2 introduces a high-resolution classifier, dimension clusters, direct location prediction, fine-grained features, and multiscale training.

#### 2.5.2. Object Detection

The backbone network generates a signature map by convolution and downsampling, and IRM-YOLOv2 divides the image into an *S* × *S* grid, where each grid is called a cell. In this study, the bulb object detection test showed that a 14 × 14 grid has lower grid computing costs and better detection accuracy than a 7 × 7 grid. Therefore, IRM-YOLOv2 uses a 14 × 14 grid.

As shown in Figure 5, IRM-YOLOv2 outputs 14 × 14 × 42 tensors (the object of this study is classified as 1), each unit predicts seven boundary boxes (equal to the number of anchor boxes) in the characteristic map, and each boundary box contains six forecast values. The six predictions are the pixel coordinates (*u_ij_*, *v_ij_*) of the upper left corner of the boundary box, width (*w_ij_*) and height (*h_ij_*), confidence scores (*p_ij_*), and class scores (*C_ij_*) of the boundary box. In model training, the definition equation for *p_ij_* is

(6) $$p_{ij}=\mathrm{Pr}\left(class_{1}\right)\cdot IOU_{pred}^{truth}\left(ij\right)$$

The value of Pr(*class*_1_) is 1 if part of the object falls in a cell and 0 otherwise. $IOU_{pred}^{truth}\left(ij\right)$ denotes the overlap between the ground truth box and the predicted bounding box. When working correctly, *p_ij_* represents the confidence of the regression. Finally, only the best bounding box is retained by non-maximum suppression (NMS) [53].

To compute the mean squared error loss between the predicted bounding boxes and the ground truth in IRM-YOLOv2, the loss function of IRM-YOLOv2 is calculated as [21,26]:

(7) $$\mathrm{Total}\;\mathrm{Loss}\;={\;\mathrm{Loss}}_{Localization}+Loss_{Confidence}+Loss_{Classification}$$

The localization loss measures the error between the predicted bounding boxes and the ground truth. The parameters for computing the localization loss include the position, size of the predicted bounding box, and ground truth.

(8) $${\mathrm{Loss}}_{Localization}=\lambda_{1}\overset{S^{2}}{\underset{i=0}{\sum }}\overset{R}{\underset{j=0}{\sum }}1_{ij}^{obj}\left[{\left(x_{i}-{\hat{x}}_{i}\right)}^{2}+{\left(y_{i}-{\hat{y}}_{i}\right)}^{2}\right]+\lambda_{1}\overset{S^{2}}{\underset{i=0}{\sum }}\overset{R}{\underset{j=0}{\sum }}1_{ij}^{obj}\left[{\left(\sqrt{w_{i}}-\sqrt{{\hat{w}}_{i}}\right)}^{2}+{\left(\sqrt{h_{i}}-\sqrt{{\hat{h}}_{i}}\right)}^{2}\right]$$

where *S* is the number of cells, *R* is the number of bounding boxes in each cell, $1_{ij}^{obj}$ is 1 if the *j*th bounding box in cell *i* is responsible for detecting the object; otherwise, $1_{ij}^{obj}$ is set to 0, $\left(x_{i},y_{i}\right)$ is the center of the *j*th bounding box in cell *i*, $\left({\hat{x}}_{i},{\hat{y}}_{i}\right)$ is the center of the ground truth in cell *i*, $w_{i}$ and $h_{i}$ are the width and height of the *j*th bounding box in cell *i*, respectively, and ${\hat{w}}_{i}$ and ${\hat{h}}_{i}$ are the width and height of the ground truth in cell *i*, respectively.

(9) $$Loss_{Confidence}=\lambda_{2}\sum_{i=0}^{S^{2}}\sum_{j=0}^{M}1_{ij}^{obj}{\left(C_{i}-{\hat{C}}_{i}\right)}^{2}+\lambda_{3}\sum_{i=0}^{S^{2}}\sum_{j=0}^{M}1_{ij}^{noobj}{\left(C_{i}-{\hat{C}}_{i}\right)}^{2}$$

where $C_{i}$ is the confidence score of the *j*th bounding box in cell *i*, and ${\hat{C}}_{i}$ is the confidence score of the ground truth in cell *i*. $1_{ij}^{noobj}$ is 1 if the *j*th bounding box in cell *i* does not contain any object; otherwise, $1_{ij}^{noobj}$ is set to 0.

(10) $$Loss_{Classification}=\lambda_{4}\sum_{i=0}^{S^{2}}1_{i}^{obj}\sum_{c\in class}{\left[q_{i}\left(c\right)-{\hat{q}}_{i}\left(c\right)\right]}^{2}$$

where $\lambda_{1}$, $\lambda_{2}$, $\lambda_{3}$, and $\lambda_{4}$ are weight values, $1_{i}^{obj}$ is 1 if an object is detected in cell *i*; otherwise, $1_{i}^{obj}$ is set to 0, $p_{i}\left(c\right)$ is the estimated conditional class probability for object class *c* in cell *i*, and ${\hat{q}}_{i}\left(c\right)$ is the actual conditional class probability for object class *c* in cell *i*.

### 2.6. Data Enhancement

**Figure 6:**
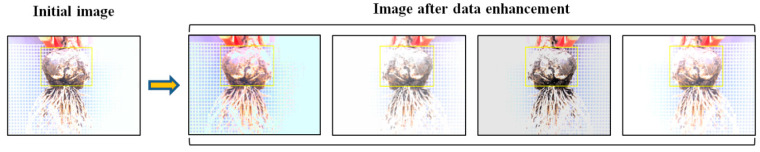
Effect of data enhancement.

Image processing techniques such as mirror image, tone, saturation, and exposure changes are used to enhance the data. To avoid serious changes in image quality caused by excessive data enhancement, each image was randomly enhanced using the above two methods. Finally, the training set was increased from 1400 images to 7000 images. Data augmentation improves the performance of the detector in training [54,55]. The data enhancements are shown in Figure 6 for the marked image, where the clay patches adhere to the bottom of the bulb. Finding bulb positions using traditional machine vision is a challenge when there is soil attached to the bulb [56].

### 2.7. Training Parameters

Model training is a key process in CNNs, and the selection of training parameters directly affects the detector performance. The important training parameters are the learning rate, small batch number, and number of training cycle epochs [37,57,58].

The learning rate determines whether and when the objective function converges to the local minimum [59]. Excessive learning rates are likely to cause value-loss explosions and sustained shocks. If the learning rate is too low, it is easy to fit, and the convergence rate of the objective function is slow. As such, this study introduces a learning rate decay mechanism in the training process; the maximum learning rate was set as 0.001, and the learning rate gradually decreased. The learning rate decay avoids excessive oscillations and facilitates the convergence of the objective function to the local minimum.

Full batch learning and online learning simultaneously use the entire training set and a single sample. In this study, mini-batch learning was used between the two. The gradient estimated by mini-batch learning deviates from the true gradient, and to make the training results robust, noise is added to the learning process [60]. Further, large minibatches cause optimization problems [61]. Appropriate minibatches introduce randomness and avoid local minima conditions [62,63]. When training with a GPU, a power of two is usually used as the minibatch size to speed up the operation. Therefore, this research compared three groups of minibatches (8, 16, and 32).

Adequate training of the model determines the performance of the detector. To compare the effects of different epochs on the training outcome, we compared three groups of epochs (30, 60, and 90).

In addition, a stochastic gradient descent mode (SGDM) training network with a momentum of 0.9 was used.

### 2.8. Model Evaluation

To better investigate the predictive ability of different detectors for non-learning objects, the performance of the detectors using the test data is used as the primary evaluation index. The evaluation indexes include the confidence score, average accuracy (AP), detection time, training time, and reliability of the cutting line position.

It should be noted that the value of AP equals the area under the precision–recall curve during model training. Confidence scores indicate both the probability of the class appearing in the box and how well the predicted box fits the object. However, neither measure the accuracy of the predicted box location.

In this study, the concept of cutting line position and reliability is proposed, where the cutting line refers to the lower line of the predicted box, and the cutting line position is predicted by the detector. $Cq_{tk}$ is defined as

(11) $$Cq_{tk}=v_{tk}+h_{tk}$$

The value of $Cq_{tk}$ is the *k*th object-detection-predicted cut-line pixel coordinates (pixels) of the *t*-algorithm.$\;Dev_{tk}$ is defined as

(12) $$Dev_{tk}=Cq_{tk}-\frac{1}{T}\sum_{t=1}^{T}Cq_{tk}$$

where $Dev_{tk}$ is the *k*th object-detection-predicted cut-line position deviation (pixel) of the *t*-algorithm. *T* is the number of algorithms used for comparison.

(13) $$SS_{t}=\sqrt{\frac{\sum_{k=1}^{Q}{\left(Dev_{tk}-\bar{Dev_{t}}\right)}^{2}}{Q-1}}$$

where $SS_{t}$ is standard deviation of the prediction deviation of the *t*-algorithm. $\bar{Dev_{t}}$ is the mean value of prediction deviation of the t-algorithm. *Q* is the number of samples.

The reliability of the cutting line position was obtained by statistical analysis of the object detection results of different algorithms, which mainly examined the standard deviation of the cutting line position and the deviations. The larger the value of $Dev_{tk}$, the farther the cutting line from the bulb and the more difficult it is to cut the bulb. The smaller the value of $SS_{t}$, the smaller the fluctuation in the object detection result. Detectors with larger deviations and smaller standard deviations were considered reliable.

## 3. Test Result

To evaluate the performance of IRM-YOLOv2 in locating bulbs, different experiments were conducted throughout this study. The system realized by the model was as follows: an NVIDIA GTX1650 (4 GB) GPU, MATLAB, and a deep neural network library (CuDNN) for GPU learning.

### 3.1. Preliminary Tests

SqueezeNet [64] and ShuffleNet [65] are representative lightweight networks. SqueezeNet [64] was accelerated and compressed by reducing the number of parameters based on the fire module structure. ShuffleNet [65] is a CNN model designed for mobile devices with limited computing power, featuring group convolution and channel-mixing operations. During training, the input of the model network was 224 × 224.

**Figure 7:**
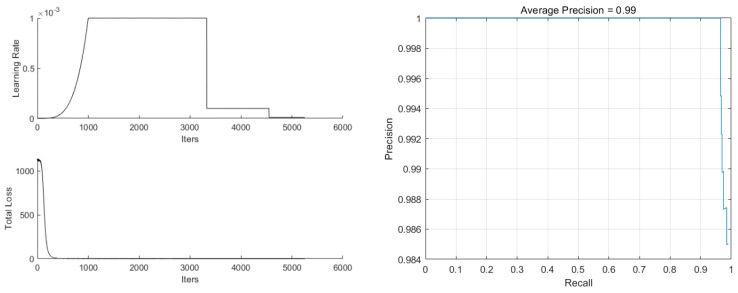
Training status of IRM-YOLOv2.

The parameters were set to minibatches of 16 and 60 epochs, and IRM-YOLOv2 was trained, as shown in Figure 7. The learning rate was gradually increased to the maximum value of 0.001 at the beginning of training and gradually decreased to 0.0001 and 0.00001 at the middle and later stages of training, respectively, which effectively avoided the oscillations of the loss function. The total loss at the end of the training was less than 0.5, and the average precision was 0.99.

**Figure 8:**
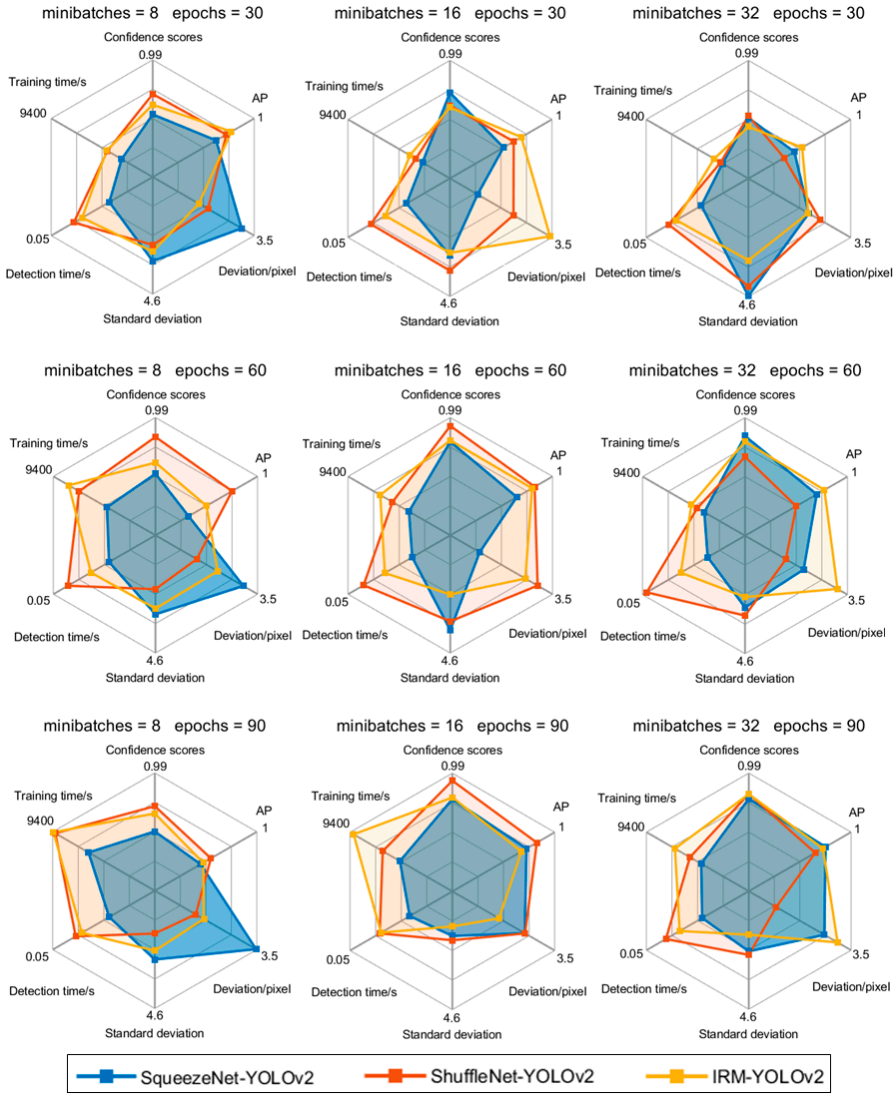
Comparison of lightweight YOLOv2 test results.

SqueezeNet-YOLOv2, ShuffleNet-YOLOv2, and IRM-YOLOv2 were trained, and the corresponding detectors were tested on the test set, as shown in Figure 8. The model still had high confidence scores on the test set, indicating that the trained detectors had not been fitted. We can see that the gradient process from the comparison, such as the deviation and standard deviation of IRM-YOLOv2, decreases with an increase in epochs. Mutant processes, such as the standard deviation of SqueezeNet-YOLOv2, first increased and then sharply decreased as epochs increased. When the minibatches were 16 and the epochs were 60 and 90, the confidence scores and APs of the three detectors were significantly higher than the other results. Evidently, the training time for 60 epochs is shorter than that with 90 epochs. Therefore, for the YOLOv2 algorithm, minibatches of 16 and 60 epochs were selected for subsequent experiments.

Although the confidence scores and APs of IRM-YOLOv2 were not the highest, the deviation of IRM-YOLOv2 was large, and the standard deviation was the smallest. This indicates that IRM-YOLOv2 has strong reliability in predicting the cutting line position. Although the confidence scores and APs of ShuffleNet-YOLOv2 were generally high, the detection time was the longest. SqueezeNet-YOLOv2 had the shortest detection time, but its standard deviation was the largest, and the variation in predicting the position of the cutting line was the largest. However, the detection time of IRM-YOLOv2 was less than 0.04 s, and object detection could be completed in a short time. A comprehensive analysis shows that the performance of IRM-YOLOv2 is better than that of SqueezeNet-YOLOv2 and ShuffleNet-YOLOv2.

### 3.2. Comparison of Algorithms

To investigate the performance of different algorithms for single-object detection, YOLOv2, YOLOv3, and YOLOv4 were selected for comparison. Eight models based on the three algorithms were compared. In this study, three classic networks, ResNet50 [49], GoogLeNet [66] and AlexNet [67] were also compared. The parameters of the YOLOv2, YOLOv3, and YOLOv4 models are presented in Table 1. YOLOv3 and YOLOv4 chose minibatches of eight because this reduced the calculation cost. It was found that the detector reached maximum recognition accuracy when trained for 30 epochs.

The YOLOv3 and YOLOv4 algorithms use multiscale feature fusion for object detection. They are mainly used for multi-object recognition and have high detection accuracy for small objects. YOLOv3-tiny and YOLOv4-tiny are the lightweight versions of YOLOv3 and YOLOv4, respectively. They are lightweight and operate smoothly when deployed on edge-computing devices. The YOLOv3-tiny architecture consists of a series of convolution and max pooling layers.

**Figure 9:**
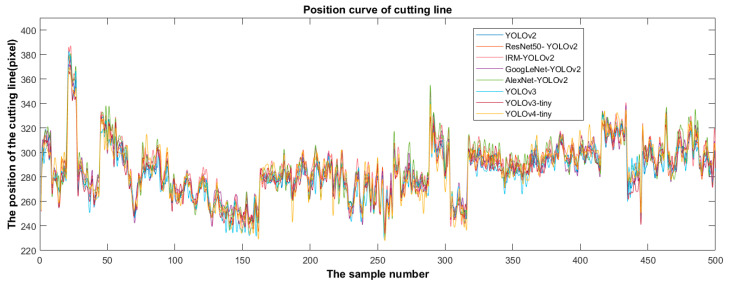
Distribution of detection results of different algorithms.

After the training process, the loss of the eight detectors before the end of training was less than 0.5, and the confidence scores were very high, indicating that the detector had not been fitted. Figure 9 shows the position distribution of the cutting line predicted by the eight detectors in the test set.

**Figure 10:**
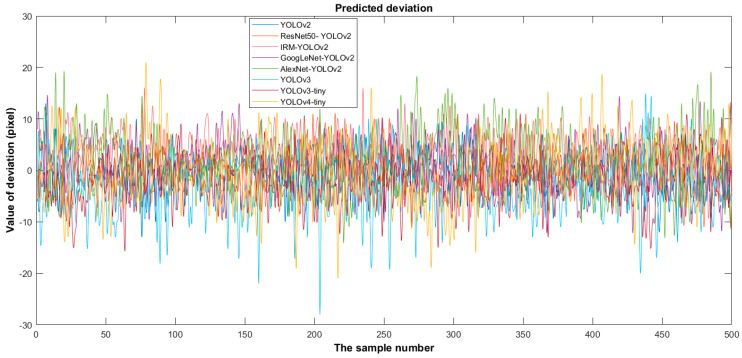
Deviation distribution of detection results of different algorithms.

Based on the predicted position distribution of the cutting line, the deviation of the eight detectors was calculated, as shown in Figure 10. Figure 10 shows that the overall trend of the cutting line position predicted by each detector is consistent according to the different test data images. The cutting-line positions were distributed in the interval of 225–390 pixels. The deviation is calculated using (12) according to the position of the cutting line predicted by the detector. The predicted deviation represents the deviation between the position of the cutting line predicted by the detector and the predicted mean value of the eight detectors. The deviation was visualized using a line diagram.

The YOLOv4-tiny fluctuation was the most obvious, with large fluctuations in both the positive and negative deviation directions. Next was AlexNet-YOLOv2, with large fluctuations in the positive deviation direction. YOLOv3 fluctuated significantly in the negative deviation direction, with a mean deviation of −3.3. Fluctuations in GoogLeNet-YOLOv2 occurred in both the positive and negative deviation directions, with a mean deviation of 0.71. YOLOv3-tiny had a certain amplitude fluctuation in the negative deviation direction, and the mean deviation was -1.99. IRM-YOLOv2, YOLOv2, and ResNet50-YOLOv2 fluctuated slightly. However, the mean IRM-YOLOv2 deviation was 2.82. This indicates that the cutting line predicted by IRM-YOLOv2 was far from the bulb, which effectively prevented the bulb from being damaged. Combining the largest deviation with the smallest standard deviation, IRM-YOLOv2 was the most reliable of the eight models.

**Figure 11:**
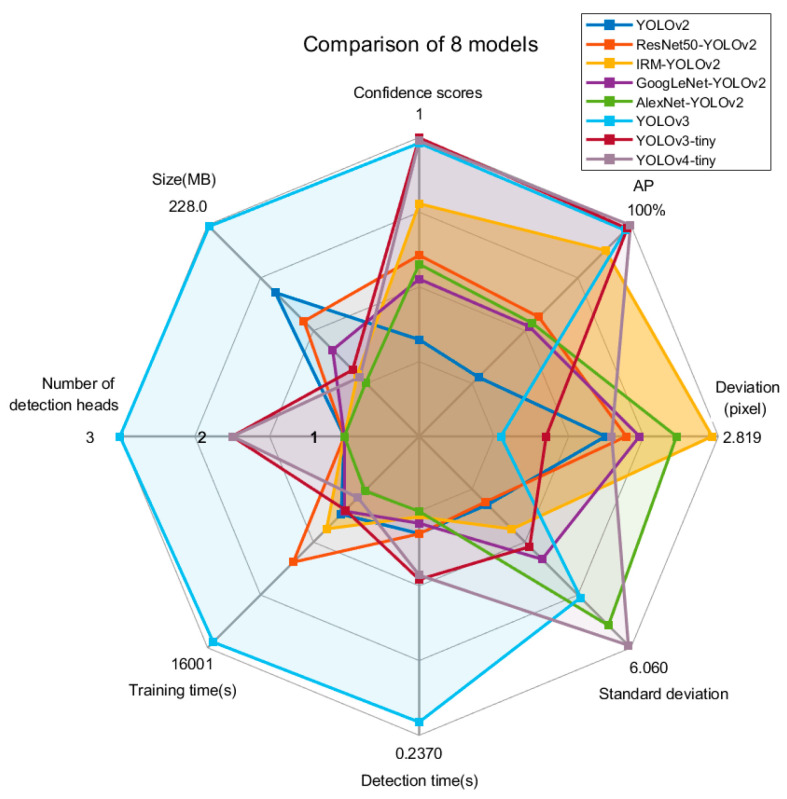
Detection performance of different algorithms.

Comparing the eight models, the YOLOv3 algorithm with multiscale feature fusion had the highest confidence score and AP. However, the YOLOv3 and YOLOv4 algorithms had a long detection time, the prediction bias fluctuated sharply, and the standard deviation of the prediction bias was the largest, indicating poor reliability. YOLOv2 and ResNet50-YOLOv2 had the smallest fluctuations in prediction deviation and the smallest standard deviation of prediction deviation but had lower confidence scores and APs, which makes accurate detection difficult. GoogLeNet-YOLOv2 and AlexNet-YOLOv2 had not only lower confidence scores and APs but also relatively poor predictive reliability. IRM-YOLOv2 considers the confidence scores, APs, and reliability. It also had a short detection time, small model space, and is easy to deploy on edge computing devices. The results are compared in Figure 11. The confidence score of IRM-YOLOv2 was 0.98228, AP was 99.2%, deviation was 2.819, standard deviation was 4.153, detection time was 0.0356 s, and the model space was 24.2 MB.

**Figure 12:**
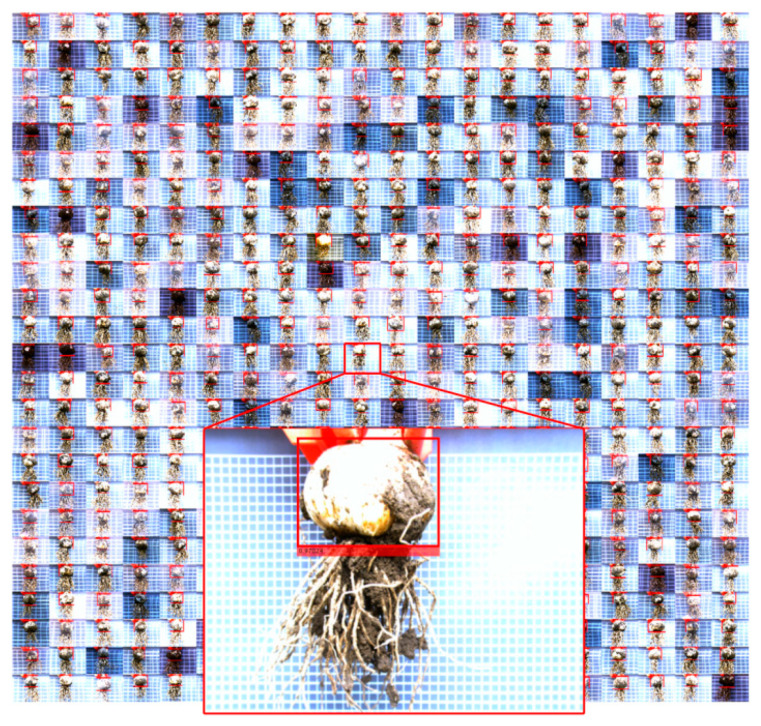
Results of the IRM-YOLOv2 detector on the test set.

The results of the bulb test on the test data obtained using IRM-YOLOv2 are shown in Figure 12. It can be seen that the detection success rate of IRM-YOLOv2 was 100% regardless of the image brightness.

**Figure 13:**
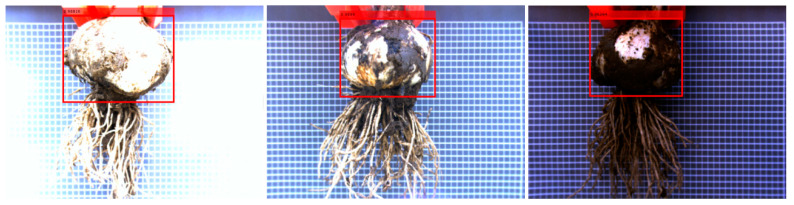
Detection results of IRM-YOLOv2 detector.

The IRM-YOLOv2 test results are shown in Figure 13. The test results show that the proposed IRM-YOLOv2 has excellent detection performance. First, IRM-YOLOv2 can operate normally under different ambient brightness conditions without any pretreatment, which reduces the detection cost and speed. Second, the adhered soil at the bottom of the bulb will not affect the detection performance of IRM-YOLOv2, and IRM-YOLOv2 can still achieve correct and fast detection. Finally, IRM-YOLOv2 is an intelligent method for detecting the location of bulb cutting lines of different shapes. Therefore, IRM-YOLOv2 meets the requirements of accuracy, speed, and reliability for garlic root cutting.

### 3.3. Visualization of Convolution Layers

**Figure 14:**
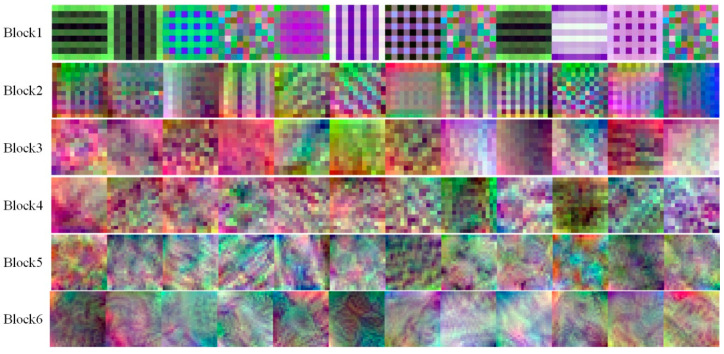
Visualization of some filters of the last convolutional layer of each block of the IRM-YOLOv2 structure.

The CNN model utilizes different convolution learning objective characteristics in the training process, which determine the final accuracy. Visualizing the features learned by the CNN during the training process will clarify the learning content of the different convolutions [7]. The convolution layer can be considered a composite filter consisting of individual filters in each dimension. To identify the characteristics learned by IRM-YOLOv2 during training, we visualize the first 12 dimensions of the filters in some convolutions from the shallow to the deeper layers of the network, as shown in Figure 14. It can be seen that the shallow filters are mainly edge detectors and color filters, whereas the edge detectors include line detectors and non-line detectors. The deeper the layer in the network, the more complex the available filters. The learning feature is an advanced combination of shallower learning features capable of learning complex patterns and textures. The convolution visualization shows that IRM-YOLOv2 was effectively trained in this study.

## 4. Discussion

With the rapid development of CNNs, new processing methods have been developed for many problems that are difficult to solve using traditional image processing methods [56]. A CNN can accurately detect an object by learning its characteristics by using a powerful neural network. Concurrently, CNNs are more robust than traditional image-processing methods. Traditional image processing methods have strict requirements regarding the brightness of the image and the illumination conditions of the object, which often increase the equipment cost. However, CNNs can learn image features under different conditions owing to the powerful learning ability of neural networks, which are suitable for scenes with large environmental differences. CNNs reduce the equipment cost, and the detection accuracy is not lower than that of traditional image processing methods.

The results of the previous study show that the detection performance of ResNet50-YOLOv2 was better than that of ResNet50-Faster R-CNN and ResNet50-SSD [33]. Through this study, the comparison of the results shows that the detection performance of IRM-YOLOv2 is better than ResNet50-Faster R-CNN and ResNet50-SSD. In the study of identifying cocoa beans, a comparison of a deep computer vision system with a conventional computer vision system revealed that the former was more accurate [70]. Studies to detect pesticide residue levels have shown that deep learning outperforms traditional machine learning [71]. In a study of barley flour classification, machine learning methods were used to show superior predictive power compared to computer vision systems [72]. The use of computer vision-based image classification methods requires the construction of a consistent image acquisition environment, which limits the usage scenarios [73]. In contrast, the use of training data including different influencing factors in this study eliminates the effects of luminance variations and differences in the amount of cover, making the IRM-YOLOv2 model robust.

**Figure 15:**
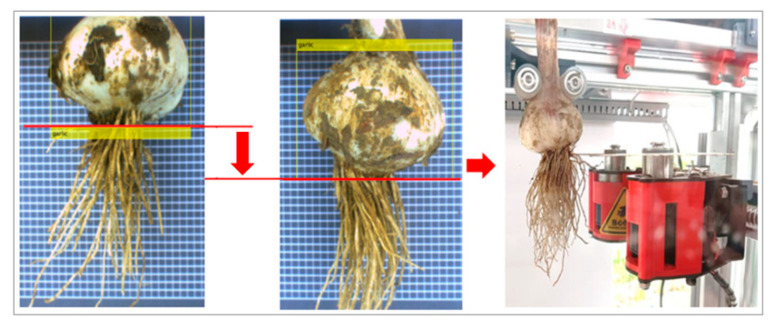
Schematic diagram of garlic root cutting controlled by IRM-YOLOv2 algorithm.

When introducing a CNN into a food processing system, the performance of the actuator in the system must also be considered. In future work, a CNN will be used for garlic root cutting. The principle of root cutting is illustrated in Figure 15. The position of the bulbs is detected by IRM-YOLOv2, and then the root cutter is moved to the corresponding cutting line position. The entire system was automatically controlled using a computer. This study investigated the efficient detection of bulb objects. Additionally, in future work, further consideration will be given to the speed and reliability of the execution mechanism.

The prediction frame of the object detection using CNN-based detection is rectangular, resulting in a straight line for the cutting line. The detection method and principles proposed in the study are not applicable if the root cutting device is required to completely remove the garlic root. However, how to cut more cleanly is the next step of the study and will be very interesting.

## 5. Conclusions

This study used machine vision combined with a CNN to detect bulbs. Based on the excavated garlic images, an optimized YOLOv2 algorithm model using an inverted residual module was established. The best minibatches and epochs were selected through parameter comparison. The results showed that the average accuracy of IRM-YOLOv2 was 99.2%, and the confidence scores, deviation, standard deviation, and detection time of IRM-YOLOv2 were 0.98228, 2.819 pixels, 4.153, and 0.0356 s, respectively.

In this study, a lightweight bulb detection method for single object detection based on machine vision combined with a CNN was proposed, which achieved good results in bulb classification and positioning and obtained better performance than the YOLOv2 algorithm and YOLOv3 and YOLOv4 algorithms for multi-object detection. The cutting line position was predicted by the CNN. The YOLO algorithm is expected to have tremendous potential for application in industrial automated food processing. IRM-YOLOv2 was robust to images of varying brightness owing to its excellent learning ability. In addition, it has high detection accuracy, fast detection speed, high reliability, and low calculation cost. Therefore, deploying IRM-YOLOv2 ensures high efficiency and low cost.

## Figures and Tables

**Table 1:** Configuration of the proposed detector model.

Model	Minibatches	Epochs	Maximum Learning Rate
YOLOv2 [26]	16	60	0.001
ResNet50-YOLOv2 [49]			
IRM-YOLOv2			
GoogLeNet-YOLOv2 [66]			
AlexNet-YOLOv2 [67]			
YOLOv3 [68]	8	30	
YOLOv3-tiny			
YOLOv4-tiny [69]			

## Data Availability

The data collected in this research are available when required.
